# Pullout strength of reinserted pedicle screws using the previous entry point and trajectory

**DOI:** 10.1186/s13018-019-1240-8

**Published:** 2019-07-04

**Authors:** Xuanwu Huang, Zixiang Huang, Liwei Xu, Dongzhu Liang, Meichao Zhang, Hui Zhang

**Affiliations:** 1 Department of Orthopedics, Zhujiang Hospital, Southern Medical University, No. 253, Gongye Road, Haizhu District, Guangzhou, 510282 China; 2 The Second School of Clinical Medicine, Southern Medical University, No. 253, Gongye Road, Haizhu District, Guangzhou, 510282 China; 3 Department of Spine Surgery, Guangdong Second Provincial General Hospital, No. 466, Xingang Road, Haizhu District, Guangzhou, 510317 China; 4 Guangdong Provincial Key Laboratory of Medical Biomechanics, Department of Anatomy, Southern Medical University, Guangzhou, China

**Keywords:** Pedicle screw, Reinserted screw, Pullout strength, Lumbar spine, In vitro study

## Abstract

**Purpose:**

This study compared the biomechanics of reinserted pedicle screws using the previous entry point and trajectory with those of correctly inserted pedicle screws.

**Methods:**

The study used 18 lumbar vertebrae (L1–6) from three fresh calf spines to insert 6.5 × 40-mm pedicle screws. A control screw was inserted correctly along the axis of one pedicle, while an experimental screw was reinserted completely using the previous entry point and trajectory in the other pedicle. The experimental screw was removed after being completely inserted in group A and after 80% of the total trajectory inserted in group B. And the experimental screw was removed after 60% of the total trajectory was reached in group C. The biomechanical values of the pedicle screws were measured.

**Results:**

There were no significant differences in pedicle screw axial pullout strength between reinserted screws and correct screws in the 3 groups (*P*_A_ = 0.463, *P*_B_ = 0.753, *P*_C_ = 0.753). Stiffness measurement increased for the reinserted screw compared with that of the control screw. Fracturing was observed between the vertebral body and pedicle.

**Conclusion:**

Theoretically, a surgeon can remove the pedicle screw when necessary, inspect the trajectory, and reinsert the screw using the previous entry point and trajectory.

## Introduction

Pedicle screw instrumentation is considered standard for treatment of spinal degenerative diseases, fractures, tumors, and deformities [1–3]. Although pedicle screw instrumentation is strong, malposition of a screw, resulting in a lateral wall breach and shorter screw insertion length in the pedicle, occurs frequently, especially with the freehand technique [4, 5]. The rate of pedicle screw malposition ranges from 5 to 41% [6, 7]. When pedicle screw malposition occurs during surgery, the screw is removed and reinserted along a correct entry point and trajectory. What is more, if a shorter pedicle screw is used because of insufficient preoperative preparation, the surgeon may remove the pedicle screw and reinsert a longer pedicle screw.

Many studies reported that the biomechanical strength of a redirected pedicle screw is less than that of a correctly placed screw [5, 8–11]. Brasiliense et al. [8] compared the pullout strength of 3 cortical perforation pedicle screws with that of standard screws. They found that lateral wall breach screws had 21% less mean pullout strength than well-placed pedicle screws. The pullout force of airball pedicle screws was 33% less than that of standard screws. Goda et al. [10] reported that after a lateral wall breach, the pullout strength of a redirected pedicle screw was 24% less than that of a standard screw. The average pullout strength of a screw that perforated the end-plate but was not removed was not significantly different from that of a standard screw. However, no study has reported the biomechanical strength of pedicle screws inserted to various depths, followed by removal and reinsertion using the previous entry point and trajectory.

Wilke et al. [12] measured bone mineral density (BMD; 1.66 ± 0.12 g/cm^3^), range of motion (2.8–5.0° in flexion and extension, 1.4° in axial rotation, 3.9–6.6° in lateral rotation), neutral zone (14–30%), and stiffness (0.07–0.38) in 12 calf spines and found that they were similar to those of human spines. They suggested that calf spines could be used to assess spinal implants as an alternative to use of human cadavers.

The purpose of this research was to compare the biomechanics of reinserted pedicle screw using the previous entry point and trajectory with that of correctly inserted pedicle screws using calf lumbar vertebrae.

## Materials and methods

This study used 18 lumbar vertebrae (L1-6) from 3 fresh calf spines (age about 6–9 months). Due to similar BMD, un-mature calf models were used. Muscles, ligaments, and intervertebral discs were removed, preserving only normal osseous structures. The vertebrae were stored at − 20 °C until the day before testing and thawed at room temperature. The vertebrae were radiographed in the anterior-posterior and lateral planes to exclude fracture, tumor, and congenital disease. And the length and diameter of pedicles were measured by a ruler.

**Fig. 1:**
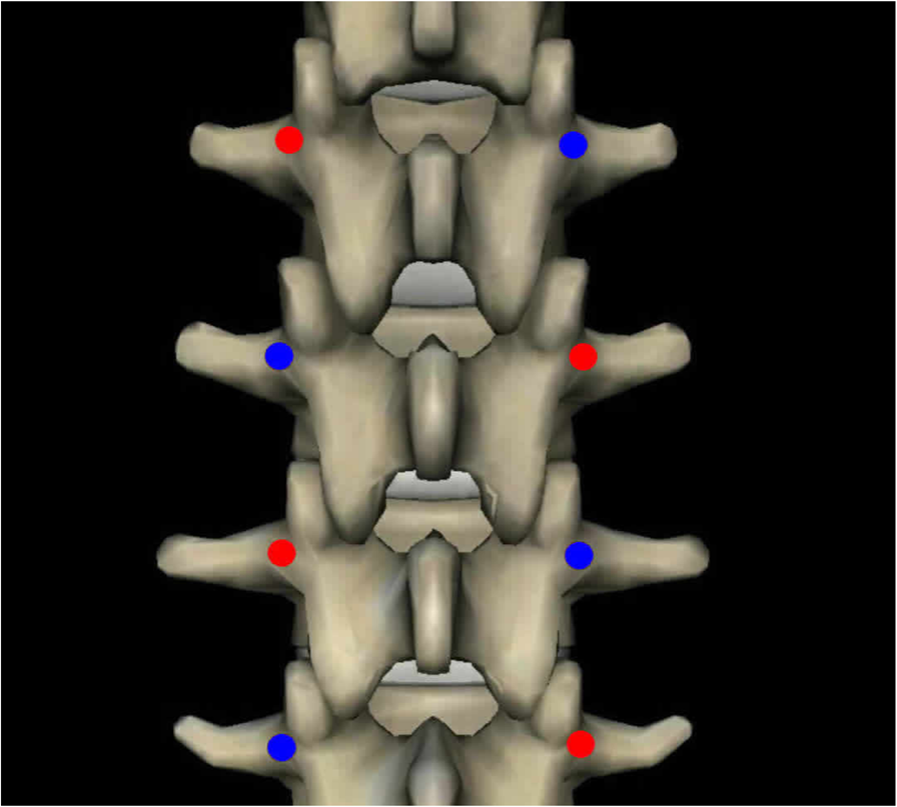
The red point is the side of experimental screw, and the blue point is the side of control screw

Before testing, the specimens were divided randomly into 3 groups according to the lumbar sequence. A control screw was inserted correctly along the axis of one pedicle, while an experimental screw was reinserted in the other pedicle, using the previous entry point and trajectory. Each screw was inserted using the same depth and angle. If a control screw was inserted in the right pedicle, a control screw would be inserted in the left pedicle of the next vertebra, so that the screws intersected (Fig. 1). The control pedicle screw was labeled with a clip.

Each pedicle screw was inserted using the freehand technique under direct visualization [2]. The entry point is similar to that of the human lumbar spine and is located at the intersection between the lateral margin of the superior articular process and the midline of the transverse process. After entry point and angle preparation, a 5.5-mm tap was inserted to enlarge the trajectory. Monoaxial pedicle screws (6.5 × 40-mm, SINO 6.0 Spine Fixation System, WEGO, Shandong, China) were then inserted. X-ray was used during the procedure to ensure proper trajectory and consistent depth. If the lateral wall or end-plate was breached, the vertebra was excluded.

**Fig. 2:**
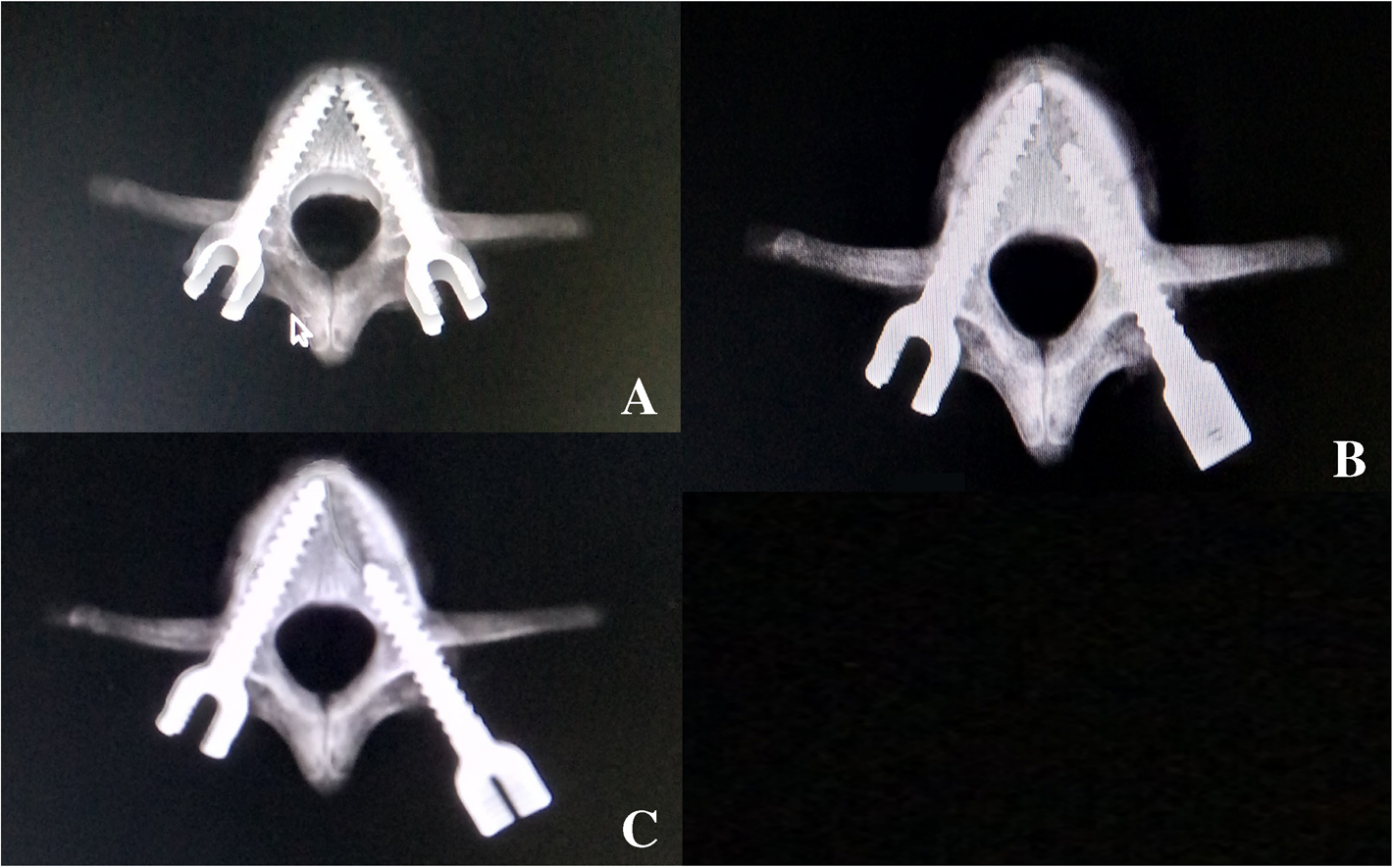
Radiographs identifying the depth of insertion. **a** Experimental screw was removed after being completely inserted in one pedicle and was then reinserted completely. **b** Experimental screw was removed after 80% of the total trajectory was reached and then reinserted completely. **c** Experimental screw was removed after 60% of the total trajectory was reached and then reinserted completely

In group A, the experimental screw was removed after being completely inserted in one pedicle and then reinserted completely using the previous entry point and trajectory after inspecting the trajectory. In group B, the experimental screw was removed after 80% of the total trajectory was reached and was then reinserted completely. In group C, the experimental screw was removed after 60% of the total trajectory was reached and was then reinserted completely (Fig. 2).

**Fig. 3:**
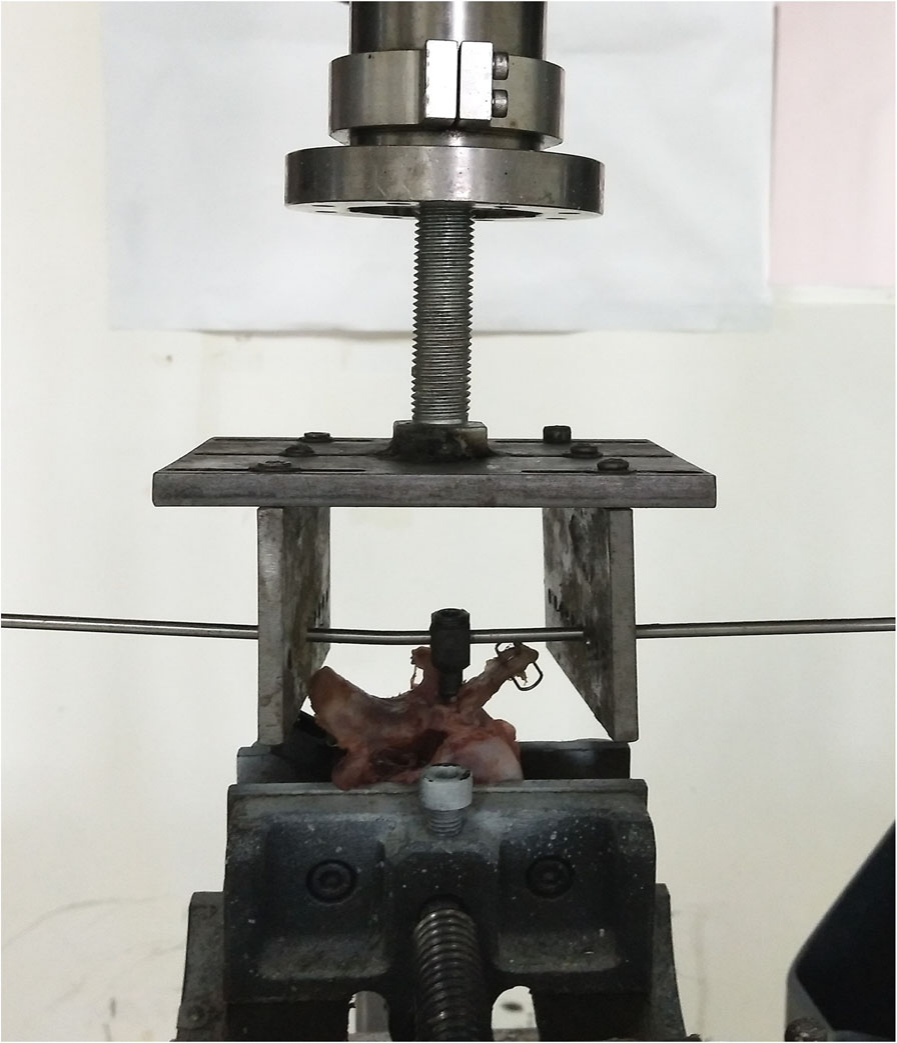
Photograph of pedicle screw pullout testing

**Fig. 4:**
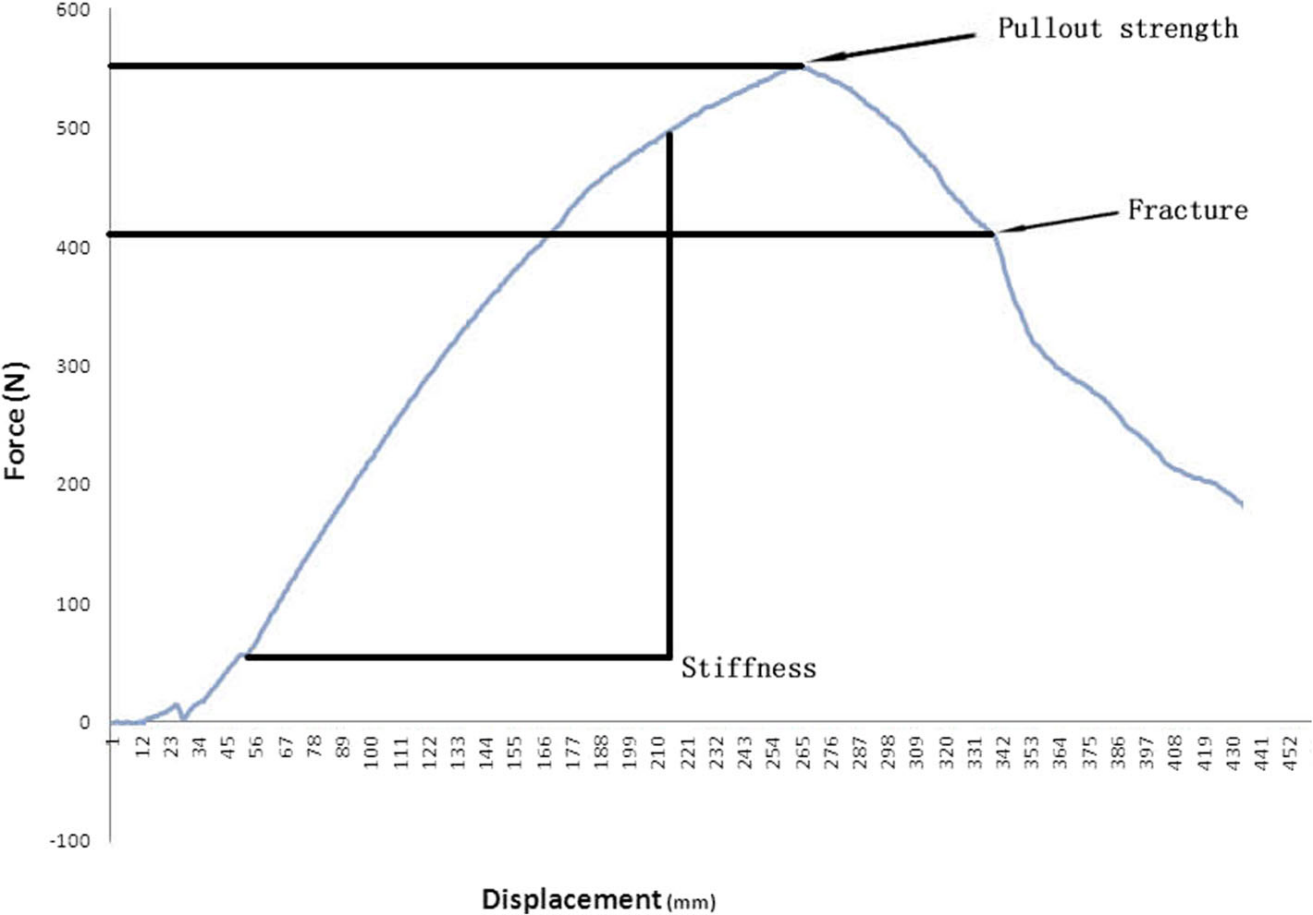
Force-displacement curve

For each pedicle screw, we assessed biomechanical properties using a material testing machine (ELF-3510AT, Bose Inc., MN, USA), with the screw pulled out along its long axis (Fig. 3) and determined the force-displacement curve [7, 11]. The pullout strength was defined as the maximum load and the stiffness was the initial slope of the force-displacement curve (Fig. 4).

All analyses were performed using SPSS 19.0 software (SPSS Inc., Chicago, IL, USA). Differences in biomechanical properties were analyzed using the paired Wilcoxon rank sum test. Significance was set for a *P* value < 0.05.

## Results

**Fig. 5:**
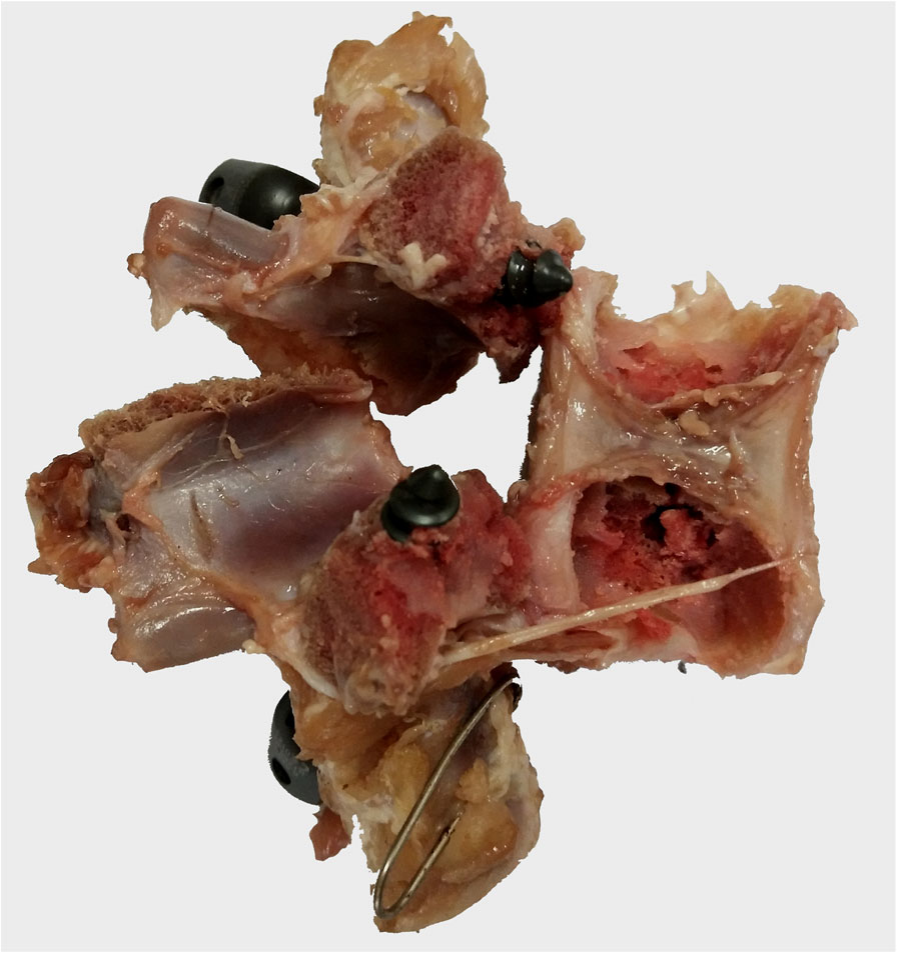
Photograph of fracture between vertebral body and pedicle

As shown in Table 1, the pullout strength was not significantly greater with use of corrected screws than with reinserted screws in the 3 groups. In group A, the mean axial pullout strength of reinserted screws was 3.7% less than that of control screws. In group B, the pullout strength of reinserted screws was 2.5% less than that of control screws. But in group C, the reinserted screws were superior in average pullout strength. Stiffness was also an important outcome. In the 3 groups, the stiffness value of reinserted screws was greater than that of control screws. There was a significant difference in group A and C but no significant difference in group B (Table 2). When the terminal end of the pedicle screw was removed through the edge of the vertebral body, a fracture developed between the vertebral body and pedicle in all samples (Fig. 5), and the load decreased more rapidly. There was no significant difference in the load associated with fracture (Table 3).

**Table 1:** Mean pullout strength

Group	Control (N)	Reinserted (N)	*P* value
A	593.47 ± 108.24	571.61 ± 106.66	0.463
B	487.99 ± 130.81	476.06 ± 186.28	0.753
C	502.72 ± 111.50	515.91 ± 161.50	0.753

**Table 2:** Mean stiffness

Group	Control (N/mm)	Reinserted (N/mm)	*P* Value
A	166.36 ± 17.19	216.61 ± 65.60	0.046
B	280.18 ± 49.58	287.45 ± 47.18	0.753
C	319.11 ± 60.54	384.13 ± 86.03	0.028

**Table 3:** Mean load of fracture

Group	Control (N)	Reinserted (N)	*P* value
A	421.65 ± 116.39	409.22 ± 88.15	0.753
B	370.34 ± 99.19	347.08 ± 129.81	0.753
C	411.77 ± 109.41	367.97 ± 99.82	0.116

## Discussion

Pedicle screw instrumentation is often used to treat spinal disease. Most surgeons insert pedicle screws using the freehand technique. Malposition of pedicle screws is a major complication during screw insertion. The reported rate of pedicle screw malposition is 5–41% [2, 6, 7].

After a pedicle screw breaches the lateral wall, the nerve can be damaged by bone chips. To avoid this situation, some surgeons remove the pedicle screw after passing through the posterior vertebral body. If the pedicle lateral wall is in good condition, the screw can be reinserted using the previous entry point and trajectory. Moreover, if a shorter pedicle screw is used because of insufficient preoperative preparation, the surgeon may remove the pedicle screw and reinsert a longer pedicle screw. Many studies have examined the biomechanics of pedicle screws [8–10, 13, 14]. However, the studies did not evaluate the pullout strength of screws that were partly inserted, backed out, and then reinserted using the same entry point and trajectory. Our study measured the pullout strength of reinserted screws using the same entry point and trajectory following removal after insertion to various depths.

BMD shows a high correlation with pedicle screw axial pullout force [15, 16]. In osteoporotic patients, the pullout strength is less than in normal persons and the pedicle screw often loosens [14, 17]. BMD has been examined in cadaveric studies using human spines. However, we compared the pullout strength of pedicle screws on two sides. Theoretically, the effect of bone mass was excluded. Therefore, we did not measure BMD.

The results indicated that there was no significant difference in pedicle screw axial pullout strength between reinserted and correctly inserted screws in the 3 groups. Second, stiffness increased in the reinserted screw compared with that in the control screw. Third, fracture occurred between the vertebral body and pedicle.

The purpose of pedicle screw instrumentation is to increase the stability of the spine. Pullout strength is one of the main indexes used to evaluate stability. In the present study, the pullout strength of reinserted pedicle screws was less than that of control screws in group A and B. However, reinserted screws were stronger than control screws in group C. No group showed statistically significant differences. The question is whether a pedicle screw should be removed after insertion to inspect the trajectory or to replace it with a longer screw. The pullout strength of reinserted screws was only slightly less than that of correctly inserted screws. Accordingly, the pedicle screw can be removed if necessary. Maeda et al. [18] found that the pullout strength of larger redirected screws after pedicle lateral wall breach was 46.9% greater than that of correctly inserted screws. Hence, if a pedicle screw must be reinserted, for example, in pedicle lateral wall breach, a screw of larger diameter is a good choice. However, the risk of pedicle wall violation will be increased using a larger diameter screw.

Pedicle screw loosening is a frequent complaint [19]. Stiffness is defined as the ability of an object to resist deformation, and predicts the initial holding capacity of the pedicle screw. The greater the stiffness, the stronger the structure comprised of vertebra and screws. By comparing both sides for stiffness, the fixation strength can be predicted. In our study, when the pedicle screw was reinserted using the previous entry point and trajectory, the stiffness was greater than when correctly inserted. This was an unexpected finding. Although there was no significant difference in group B (*P* = 0.753), the results are worth noting. The reasons for these findings are unknown. It is possible that the specimen was insufficient. Further study is required. Nonetheless, we concluded that the stiffness of inserted screws did not decrease.

In every specimen, fractures developed between the vertebral body and pedicle when the terminal end of the pedicle screw was removed through the edge of the vertebral body. The vertebral body consists mainly of cancellous bone, while the major structure of the pedicle is cortical bone. Thus, fracture can easily occur at the interface. Clinically, when the pedicle screw is pulled out accidentally, the possibility of fracture should be considered, especially in the presence of osteoporosis.

A limitation of this study is that we were only able to evaluate the biomechanics of the calf spine because human spines were not available. Although Wilke et al. [12] studied the calf spine for use as a substitute for the human spine, the calf spine is still different. Additionally, muscles and ligaments were removed before testing. This limitation of an in vitro study may have affected the results. Long-term follow-up is needed in patients after reinsertion of pedicle screws, to better understand the biomechanical properties.

## Conclusion

In spite of the limitations of this study, the pullout strength was not found to be significantly greater between correctly inserted screws and reinserted screws using the previous entry point and trajectory. Theoretically, a surgeon can remove the pedicle screw if necessary, inspect the trajectory, and reinsert the screw using the previous entry point and trajectory. Despite this finding, pedicle screws should be inserted carefully and removal should be avoided as much as possible.

## Data Availability

Not applicable

## Abbreviations

BMD: Bone mineral density

## References

[1] Tian NF, Huang QS, Zhou P, Zhou Y, Wu RK, Lou Y (2011). Pedicle screw insertion accuracy with different assisted methods: a systematic review and meta-analysis of comparative studies. Eur Spine J.

[2] Perna F, Borghi R, Pilla F, Stefanini N, Mazzotti A, Chehrassan M (2016). Pedicle screw insertion techniques: an update and review of the literature. Musculoskelet Surg.

[3] Liu PY, Lai PL, Lin CL (2017). A biomechanical investigation of different screw head designs for vertebral derotation in scoliosis surgery. Spine J.

[4] Kosmopoulos V, Schizas C (2007). Pedicle screw placement accuracy: a meta-analysis. Spine..

[5] Lekovic GP, Potts EA, Karahalios DG, Hall G (2007). A comparison of two techniques in image-guided thoracic pedicle screw placement: a retrospective study of 37 patients and 277 pedicle screws. J Neurosurg Spine..

[6] Gelalis Ioannis D., Paschos Nikolaos K., Pakos Emilios E., Politis Angelos N., Arnaoutoglou Christina M., Karageorgos Athanasios C., Ploumis Avraam, Xenakis Theodoros A. (2011). Accuracy of pedicle screw placement: a systematic review of prospective in vivo studies comparing free hand, fluoroscopy guidance and navigation techniques. European Spine Journal.

[7] McGilvray KC, Waldorff EI, Easley J, Seim HB, Zhang N, Linovitz RJ (2017). Evaluation of a polyetheretherketone (PEEK) titanium composite interbody spacer in an ovine lumbar interbody fusion model: biomechanical, microcomputed tomographic, and histologic analyses. Spine J.

[8] Brasiliense LB, Theodore N, Lazaro BC, Sayed ZA, Deniz FE, Sonntag VK (2010). Quantitative analysis of misplaced pedicle screws in the thoracic spine: how much pullout strength is lost?: presented at the 2009 Joint Spine Section Meeting. J Neurosurg Spine.

[9] Stauff MP, Freedman BA, Kim JH, Hamasaki T, Yoon ST, Hutton WC (2014). The effect of pedicle screw redirection after lateral wall breach--a biomechanical study using human lumbar vertebrae. Spine J.

[10] Goda Y, Higashino K, Toki S, Suzuki D, Kobayashi T, Matsuura T (2016). The pullout strength of pedicle screws following redirection after lateral wall breach or end-plate breach. Spine..

[11] Varghese V, Saravana Kumar G, Krishnan V (2017). Effect of various factors on pull out strength of pedicle screw in normal and osteoporotic cancellous bone models. Med Eng Phys.

[12] Wilke HJ, Krischak S, Claes L (1996). Biomechanical comparison of calf and human spines. J Orthop Res.

[13] Li N, He D, Xing Y, Lv Y, Tian W (2015). The effect of lateral wall perforation on screw pull-out strength: a cadaveric study. J Orthop Surg Res.

[14] Varghese V, Ramu P, Krishnan V, Saravana Kumar G (2016). Pull out strength calculator for pedicle screws using a surrogate ensemble approach. Comput Methods Prog Biomed.

[15] Halvorson TL, Kelley LA, Thomas KA, Whitecloud TS, Cook SD (1994). Effects of bone mineral density on pedicle screw fixation. Spine..

[16] Amirouche F, Solitro GF, Magnan BP (2016). Stability and spine pedicle screws fixation strength-a comparative study of bone density and insertion angle. Spine Deform.

[17] Hoppe S, Keel MJ (2017). Pedicle screw augmentation in osteoporotic spine: indications, limitations and technical aspects. Eur J Trauma Emerg Surg.

[18] Maeda T, Higashino K, Manabe H, Yamashita K, Hayashi F, Goda Y (2018). Pullout strength of pedicle screws following redirection after lateral or medial wall breach. Spine..

[19] Galbusera Fabio, Volkheimer David, Reitmaier Sandra, Berger-Roscher Nikolaus, Kienle Annette, Wilke Hans-Joachim (2015). Pedicle screw loosening: a clinically relevant complication?. European Spine Journal.

